# Factors associated with high 24-month persistence with denosumab: results of a real-world, non-interventional study of women with postmenopausal osteoporosis in Germany, Austria, Greece, and Belgium

**DOI:** 10.1007/s11657-017-0351-2

**Published:** 2017-06-22

**Authors:** A. Fahrleitner-Pammer, N. Papaioannou, E. Gielen, M. Feudjo Tepie, C. Toffis, I. Frieling, P. Geusens, P. Makras, E. Boschitsch, J. Callens, A. D. Anastasilakis, C. Niedhart, H. Resch, L. Kalouche-Khalil, P. Hadji

**Affiliations:** 1 Department of Endocrinology and Metabolism, Medical University Graz, Graz, Austria; 2 Medical School, Laboratory for the Research of Musculoskeletal System, KAT Hospital, University of Athens, Athens, Greece; 3 Department of Geriatrics and Center for Metabolic Bone Diseases, UZ Leuven, Leuven, Belgium; 4 Amgen Ltd, Uxbridge, UK; 5 Amgen Ltd, Cambridge, UK; 6 Osteoporosis Center, Hamburg, Germany; 7 Department of Internal Medicine, Maastricht University Medical Centre, Maastricht, Netherlands; 8 University Hasselt, Diepenbeek, Belgium; 9 Department of Endocrinology and Diabetes, 251 Hellenic Airforce and VA General Hospital, Athens, Greece; 10 Ambulatorium KLIMAX, Menopause and Osteoporosis Clinic, Vienna, Austria; 11 Department of Orthopedics, Rheumatology and Physical Medicine and Rehabilitation, AZ Zeno, Knokke, Belgium; 12 Department of Endocrinology, 424 General Military Hospital, Thessaloniki, Greece; 13 Osteoporosis Center, Heinsberg, Germany; 14 Medical Department II, St Vincent Hospital, Academic Teaching Hospital of the Medical University of Vienna, Vienna, Austria; 15 Medical Faculty, Sigmund Freud University, Vienna, Austria; 16 Amgen (Europe) GmbH, Zug, Switzerland; 17 Department of Obstetrics and Gynecology, Krankenhaus Nordwest, Frankfurt, Germany

**Keywords:** Adherence, Compliance, Denosumab, Non-interventional study, Osteoporosis, Persistence

## Abstract

**Summary:**

Persistence with osteoporosis therapy is vital for fracture prevention. This non-interventional study of postmenopausal women receiving denosumab in Germany, Austria, Greece, and Belgium found that persistence with denosumab remains consistently high after 24 months in patients at high risk of fracture.

**Purpose:**

Continued persistence with osteoporosis therapy is vital for fracture prevention. This non-interventional study of clinical practice evaluated medication-taking behavior of postmenopausal women receiving denosumab in Germany, Austria, Greece, and Belgium and factors influencing persistence.

**Methods:**

Subcutaneous denosumab (60 mg every 6 months) was assigned according to prescribing information and local guidelines before and independently of enrollment; outcomes were recorded during routine practice for up to 24 months. Persistence was defined as receiving the subsequent injection within 6 months + 8 weeks of the previous injection and adherence as administration of subsequent injections within 6 months ± 4 weeks of the previous injection. Medication coverage ratio (MCR) was calculated as the proportion of time a patient was covered by denosumab. Associations between pre-specified baseline covariates and 24-month persistence were assessed using multivariable logistic regression.

**Results:**

The 24-month analyses included 1479 women (mean age 66.3–72.5 years) from 140 sites; persistence with denosumab was 75.1–86.0%, adherence 62.9–70.1%, and mean MCR 87.4–92.4%. No covariate had a significant effect on persistence across all four countries. For three countries, a recent fall decreased persistence; patients were generally older with chronic medical conditions. In some countries, other covariates (e.g., older age, comorbidity, immobility, and prescribing reasons) decreased persistence. Adverse drug reactions were reported in 2.3–6.9% patients.

**Conclusions:**

Twenty-four-month persistence with denosumab is consistently high among postmenopausal women in Europe and may be influenced by patient characteristics. Further studies are needed to identify determinants of low persistence.

**Electronic supplementary material:**

The online version of this article (doi:10.1007/s11657-017-0351-2) contains supplementary material, which is available to authorized users.

## Introduction

With an aging population, the prevalence of osteoporosis in Europe is expected to rise by approximately a quarter between 2010 and 2025, resulting in a similar increase in the incidence of osteoporotic (fragility) fracture [1, 2]. In 2010, there were 3.5 million new fragility fractures among women in the European Union (EU) [3]. Such fractures are a major cause of morbidity owing to the pain and immobility with which they are associated. Moreover, they increase mortality, with hip fractures being associated with half of the deaths attributable to osteoporosis [2]. Fragility fractures also place a considerable burden on healthcare resources. The majority of hip fractures result in hospitalization, which is often lengthy, and costs associated with osteoporosis are expected to reach €46.8 billion by 2025 [2].

Because osteoporosis is a chronic condition requiring long-term treatment, poor persistence with and adherence to medication are key issues. Adherence (also referred to as compliance) describes the proportion of doses that are taken as prescribed (and also reflects the need for the patient to begin taking the medication at all), while persistence describes the duration of time over which the treatment is taken without exceeding a pre-defined permissible gap between doses [4]. Impairments to these aspects of medicine-taking behavior in the osteoporosis setting have been associated with increased fracture risk [5–9]; hence, understanding the factors associated with suboptimal adherence and persistence has the potential to improve patient outcomes and reduce the burden placed on healthcare systems.

Until recently, bisphosphonates have been the mainstay of treatment for postmenopausal osteoporosis (PMO) and have demonstrated anti-fracture efficacy [10]. However, oral bisphosphonates, which are frequently used as a first-line treatment option, are poorly absorbed if taken with food and can be associated with gastrointestinal irritation [11, 12]. These issues can lead to low persistence with oral bisphosphonate therapy; indeed, 30% of patients or fewer remain on treatment after 2 years [5, 13–15]. Suboptimal persistence has also been reported for intravenous bisphosphonates [13].

In patients with osteoporosis, previous osteoporosis therapy and a history of fractures have been associated with better persistence, and a history of falls is associated with improved compliance [14, 16, 17]; patients with these characteristics are more likely to be better educated about their disease and the importance of continuing with treatment. Several studies have shown that younger age, specifically individuals less than 60 years old versus older age, is associated with lower persistence and adherence [13, 16, 18, 19]. While one study found no association between age and persistence [14], others have reported older age to be associated with lower persistence [17]. The presence of multiple comorbidities has been shown to be linked to low persistence and adherence. In addition, patients with poor general health and those who smoke have been shown to have low persistence [16–18, 20, 21], perhaps because, in general, such individuals are less likely to take care of their health.

Denosumab, a fully human monoclonal antibody that selectively targets the RANK ligand, is an alternative antiresorptive treatment for osteoporosis. In randomized trials, denosumab 60 mg reduced the risk of fracture compared with placebo and, compared with bisphosphonates, decreased markers of bone turnover and was associated with greater bone mineral density (BMD) increases [22–27]. In patients with osteoporosis, denosumab is administered subcutaneously (SC) every 6 months (Q6M) [28]. Several observational studies of routine clinical practice suggest that a 6- or 12-monthly dosing regimen may result in improved 12-month persistence compared with both oral bisphosphonates and quarterly intravenous regimens [13–15, 29, 30].

To gain a better understanding of real-world persistence with SC denosumab Q6M and the factors associated with persistence, we conducted a non-interventional study of routine clinical practice in Germany, Austria, Greece, and Belgium. Results of a pre-specified interim analysis showed high persistence (87.0–95.3%) and adherence (82.7–89.3%) and a high medication coverage ratio (MCR; the proportion of time a patient was covered by denosumab; 91.3–95.4%) at 12 months [31]. A similar study conducted in North America showed that persistence with denosumab was 81% at 12 months and 50% at 24 months [32, 33]. Few other studies have examined long-term persistence with denosumab treatment in clinical practice or have investigated the patient characteristics associated with higher persistence [13–15, 34]. Here, we report data from the final 24-month analysis and a multivariable analysis of factors associated with persistence.

## Methods

### Study design

This international, multicenter, prospective, non-interventional study assessed persistence, adherence, and MCR in postmenopausal women receiving denosumab in routine practice in Germany, Austria, Greece, and Belgium. Enrollment began in 2011, and the study was completed on 31 August 2015. Patients were followed up for 2 years after their first denosumab injection and were expected to receive subsequent injections every 6 months.

The methodology has been described previously [31]. In brief, planned enrollment was approximately 1500 patients across the four participating countries. To be eligible to participate in the study, patients must have been suitable for treatment with, and to have been prescribed, SC denosumab 60 mg Q6M in accordance with the appropriate prescribing information (e.g., EU Summary of Product Characteristics or the local equivalent) for the treatment of PMO and in accordance with national guidelines. Patients were ineligible for this study if they were currently enrolled in, or had been enrolled in the past 6 months in, any other study involving another procedure, device, or drug, or had any disorder that the investigator felt may affect their ability to provide informed consent.

To ensure minimal disruption to routine practice, physicians must have decided to prescribe denosumab independently of, and prior to, enrollment in the study. After the first denosumab injection, the informed consent and patient enrollment procedures were completed within 4 weeks. At enrollment, all patients completed the eight-item Morisky Medication Adherence Scale (MMAS-8) questionnaire, which measures adherent behavior on a scale of 0 to 8 [35]. All other data were collected during routine practice. There was no requirement for additional tests such as dual-energy X-ray absorptiometry. Patients were permitted to take concomitant therapies that the investigators deemed necessary. All occurrences of adverse drug reactions (ADRs; considered by the physician to be related to the drug) were to be reported to the study sponsor (Amgen Inc.) by the physician.

### Analyses

The 24-month analyses reported here were prospectively planned and were to be based on the full analysis set (FAS), defined as all enrolled women who had provided informed consent and received at least one injection of denosumab. Safety outcomes were assessed in the safety analysis set (SAS), which included all enrolled patients who received at least one injection of denosumab. At the time of the 24-month analyses, sign-off on the Investigator Verification page for one site could not be obtained; all patients from this site were excluded from the 24-month FAS and SAS analyses. One patient who had been enrolled in error (and had received one denosumab injection) was also excluded from the 24-month FAS (but not the SAS).

Persistence and adherence at 24 months were reported by country as percentages and 95% confidence intervals (CIs); MCRs at 24 months were reported by country as means and 95% CIs. Persistence was defined as receipt of the subsequent injection within 6 months + 8 weeks of the previous injection and adherence as receiving two consecutive injections within 6 months ± 4 weeks of each other. Sensitivity analyses were conducted using alternative time windows of 6 months + 4, 6, and 12 weeks for persistence and 6 months ±6, 8, and 12 weeks for adherence. The rationale for these time periods has been described previously [31]. Briefly, the definition of adherence is based on pharmacokinetic/pharmacodynamic data on the residual effects of denosumab following injection, while the definition of persistence allows for the practical delays that patients may face in arranging repeat injections [31]. The MCR was calculated using the percentage of time that a patient was covered by denosumab, as assessed from prescription records, and was based on the assumption that each injection of denosumab provides 6 months (defined as 183 days) of medication coverage.

Multivariable logistic regression was used to explore associations between pre-specified baseline covariates and 24-month persistence. For each country, a stepwise logistic regression model was used; a *p* value ≤0.25 was required for a covariate to enter the model, and a *p* value ≤0.3 was required for a covariate to remain in the model. For individual covariates, a *p* value ≤0.05 was deemed to be statistically significant. The covariates included are listed in Online Resource 1; clinically relevant covariates were defined retrospectively.

### Ethics

All sites obtained local approval in accordance with the ethical principles of the Declaration of Helsinki. Each site provided Independent Ethics Committee or Independent Review Board protocol approval to Amgen along with all documentation pertaining to each patient before being permitted to participate in the study. All patients were assured of their right to withdraw from the study at any time without prejudice.

## Results

### Study population

**Fig. 1:**
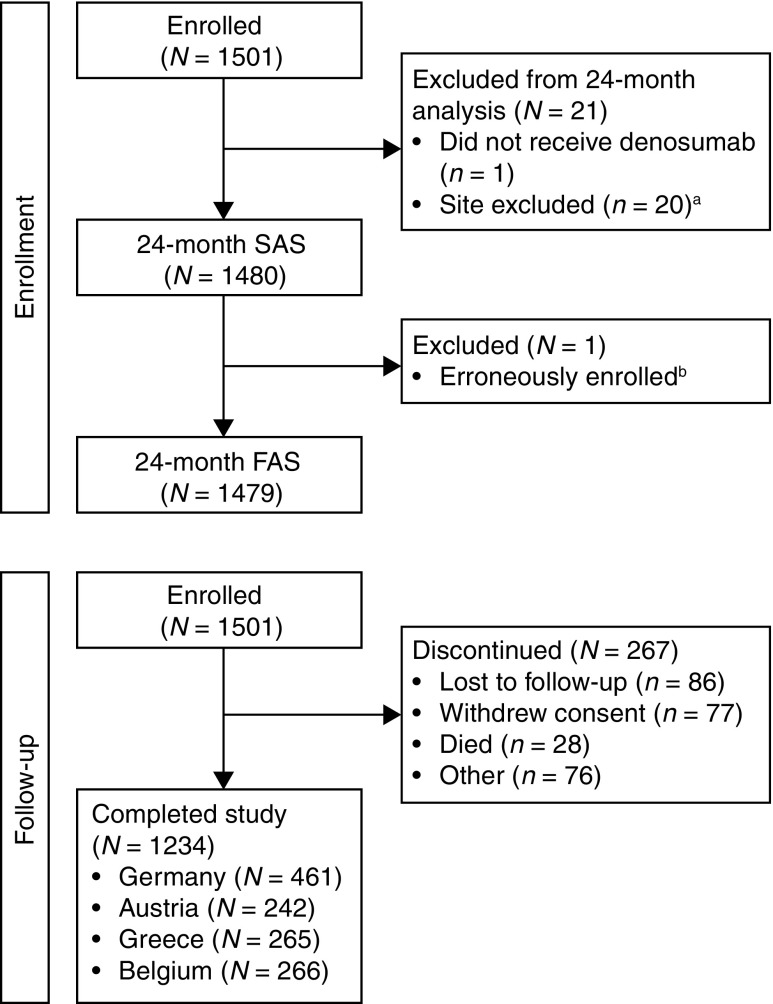
Study flow diagram. ^a^Site excluded because sign-off on the Investigator Verification Page could not be obtained. Compared with the overall population, patients at the excluded site were of similar age; had fewer comorbidities; were taking fewer concomitant medications at baseline; had a lower incidence of historical fracture, prior fall, and discontinuation of prior postmenopausal osteoporosis therapy; had higher bone mineral density T-scores at total hip; and had less severe disease. ^b^This patient received one dose of denosumab and so was included in the SAS. *FAS* full analysis set, *SAS* safety analysis set

A total of 1501 patients were enrolled in the study. Of these, 1500 were included in the FAS for the 12-month analysis [31]. Twenty patients from one site were subsequently excluded from the 24-month analysis because sign-off on the Investigator Verification Page could not be obtained; one patient enrolled in error was also excluded. Hence, a total of 1479 patients from 140 sites were included in the FAS for the 24-month analysis (Fig. 1). The 24-month FAS comprised 579 patients from Germany, 300 from Austria, 299 from Greece, and 301 from Belgium. In total, 1234 patients completed the 24-month observation period: 461 from Germany, 242 from Austria, 265 from Greece, and 266 from Belgium. The 24-month SAS comprised 1480 patients (579 patients from Germany, 300 from Austria, 300 from Greece, and 301 from Belgium).

Physician and patient characteristics for the 12-month FAS have been described previously [31]. These were generally similar across countries, although patients in Greece were younger than those in Germany, Austria, and Belgium, and were less likely to have had a prior fragility fracture [31]. Patient baseline demographics for the 24-month FAS are summarized in Table 1 and were very similar to those in the 12-month FAS, with the same differences observed between countries.

**Table 1:** Baseline demographics, comorbidities, and medical history (24 months, FAS)

Characteristic	Germany (*N* = 579)	Austria (*N* = 300)	Greece (*N* = 299)	Belgium (*N* = 301)
Age, years, mean (SD)	72.5 (8.7)	71.0 (9.5)	66.3 (9.2)	71.2 (10.4)
Time since PMO diagnosis, years, mean (SD)	6.0 (5.7)	6.5 (6.5)	6.4 (6.3)	6.4 (8.2)
Baseline T-score, mean (SD)				
Total hip	−2.1 (0.8)	−2.0 (0.8)	−2.03 (0.9)	−2.1 (0.9)
Femoral neck	−2.3 (0.9)	−2.2 (0.8)	−2.6 (0.8)	−2.5 (0.7)
Lumbar spine	−2.7 (1.1)	−2.8 (0.9)	−2.7 (0.8)	−2.2 (1.3)
Smoking status, *n* (%)				
Never	407 (70.3)	212 (70.7)	228 (76.3)	236 (78.4)
Former	52 (9.0)	40 (13.3)	24 (8.0)	32 (10.6)
Current	50 (8.6)	48 (16.0)	47 (15.7)	33 (11.0)
Missing	70 (12.1)	0 (0.0)	0 (0.0)	0 (0.0)
Any chronic medical condition^a^, *n* (%)	522 (90.2)	250 (83.3)	243 (81.3)	210 (69.8)
Modified Wolfe comorbidity index, median (Q1, Q3)	1.0 (0.0, 2.0)	1.0 (0.0, 2.0)	1.0 (0.0, 2.0)	1.0 (0.0, 3.0)
Prior fragility fracture, *n* (%)	366 (63.2)	122 (40.7)	92 (30.8)	151 (50.2)
Vertebral	180 (31.1)	28 (9.3)	34 (11.4)	52 (17.3)
Non-vertebral	256 (44.2)	101 (33.7)	65 (21.7)	126 (41.9)
Hip	19 (3.3)	11 (3.7)	13 (4.3)	25 (8.3)
Previous PMO therapy, *n* (%)	513 (88.6)	252 (84.0)	244 (81.6)	256 (85.0)
Previous PMO therapy in the 12 months before enrollment, *n* (%)	447 (77.2)	232 (77.3)	216 (72.2)	222 (73.8)
History of discontinuation of PMO therapy (other than calcium and vitamin D), *n* (%)				
Yes	102 (17.6)	59 (19.7)	47 (15.7)	44 (14.6)
No	334 (57.7)	226 (75.3)	215 (71.9)	217 (72.1)
N/A	138 (23.8)	15 (5.0)	37 (12.4)	40 (13.3)
Missing	5 (0.9)	0 (0.0)	0 (0.0)	0 (0.0)
MMAS-8				
Total score^b^, mean (SD)	7.0 (1.3)	6.6 (1.5)	6.2 (1.8)	7.1 (1.6)
Low or medium score, *n* (%)	261 (45.1)	177 (59.0)	186 (62.2)	113 (37.5)

*FAS* full analysis set, *MMAS-8* 8-item Morisky Medication Adherence Scale, *N/A* not applicable, *PMO* postmenopausal osteoporosis, *Q* quartile, SD standard deviation

^a^Comprising diabetes, osteoporosis, and hypertension

^b^Scores calculated from women who answered all questions in the MMAS-8 questionnaire. Scores ranged from 0 to 8, with high adherence represented by a score of 8, medium adherence by a score of 6–7, and low adherence by a score of less than 6

### Persistence, adherence, and MCR at 24 months

**Fig. 2:**
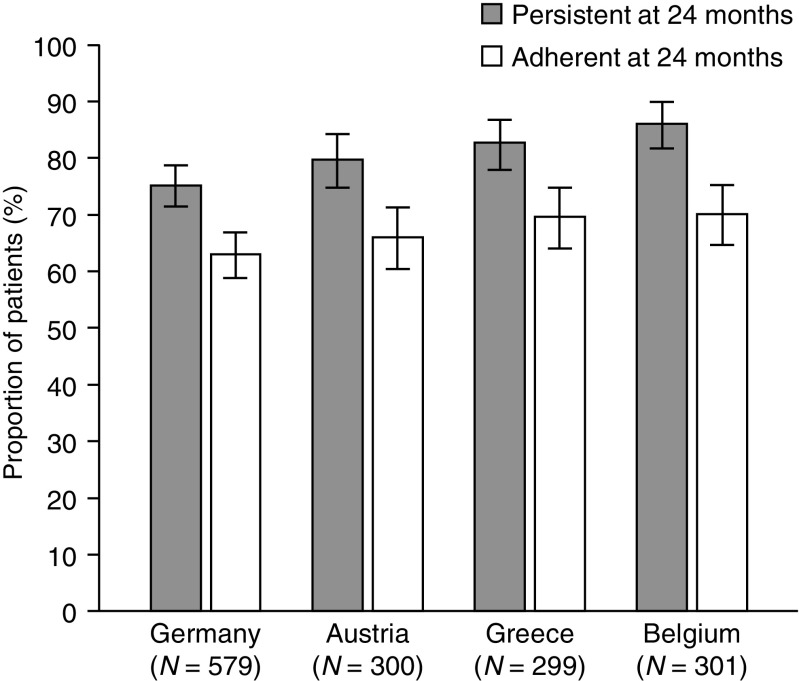
Persistence with and adherence to denosumab treatment at 24 months. Data are shown as percentages ±95% CIs. Persistence was defined as receiving the subsequent injection within 6 months + 8 weeks of the previous injection. Adherence was defined as administration of the subsequent injection within 6 months ± 4 weeks of the previous injection. *CI* confidence interval

Across the four participating countries, 75.1–86.0% of patients received a fourth denosumab injection within 6 months + 8 weeks of the third injection and were therefore classified as being persistent with treatment at 24 months (Fig. 2). Adherence at 24 months (i.e., the proportion of patients who had received a fourth denosumab injection within 6 months ± 4 weeks of the third injection) was 62.9–70.1% (Fig. 2) and the mean MCR was 87.4–92.4% (Online Resource 2). Sensitivity analyses confirmed these results (Online Resource 3).

### Multivariable analysis of factors associated with 24-month persistence

**Fig. 3:**
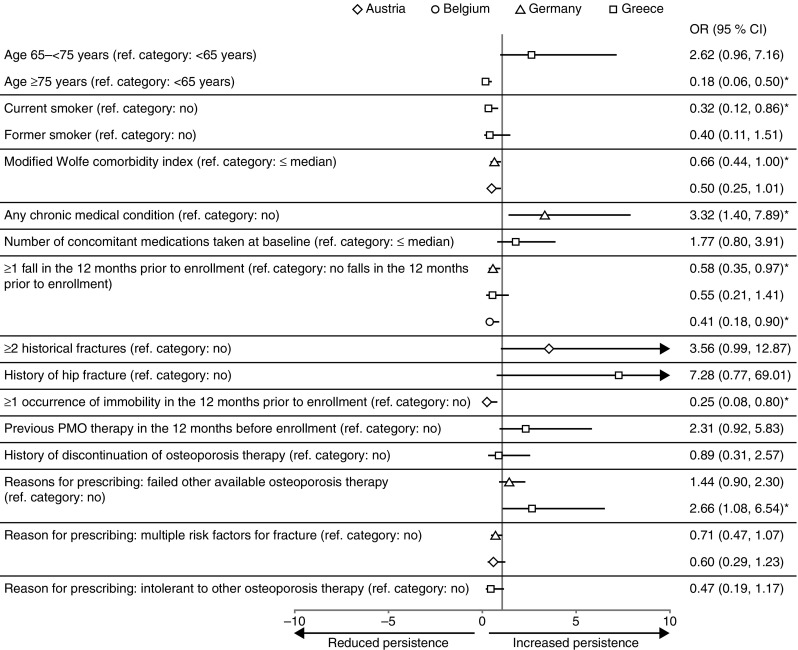
Multivariable analysis of factors associated with 24-month persistence with denosumab treatment. *p ≤ 0.05. This graph has been cropped; *arrows* indicate where error bars exceed the axis limits. *CI* confidence interval, *OR* odds ratio, *PMO* postmenopausal osteoporosis

The results of the analysis for all pre-specified covariates are presented in Online Resource 1. Covariates deemed to be clinically relevant are shown in Table 2 and Fig. 3 and are discussed herein. Given the small number of patients who were not persistent with treatment at 24 months (299 of 1479 patients), the data described below should be interpreted with caution.

**Table 2:** Summary of multivariable analysis of persistence with denosumab treatment at 24 months for covariates deemed to be clinically significant

Covariate	Germany (*N* = 579)	Austria (*N* = 300)	Greece (*N* = 299)	Belgium (*N* = 301)
	*n*/*N1* (%)	*n*/*N1* (%)	*n*/*N1* (%)	*n*/*N1* (%)
Age				
<65 years (reference category)	–	–	110/133 (82.7)	–
65–<75 years			95/103 (92.2)	
≥75 years			40/59 (67.8)***	
Current smoker				
No (reference category)	–	–	210/248 (84.7)	–
Yes			35/47 (74.5)*	
Former smoker				
No (reference category)	–	–	226/271 (83.4)	–
Yes			19/24 (79.2)	
Modified Wolfe comorbidity index				
≤Median (reference category)	242/316 (76.6)	128/158 (81.0)	–	–
>Median	169/237 (71.3)*	98/128 (76.6)		
Any chronic medical condition				
No (reference category)	16/28 (57.1)	–	–	–
Yes	395/525 (75.2)**			
Number of concomitant medications taken at baseline				
≤Median (reference category)	–	–	134/164 (81.7)	–
>Median			111/131 (84.7)	
≥1 fall in the 12 months prior to enrollment				
No (reference category)	341/452 (75.4)	–	210/247 (85.0)	201/226 (88.9)
Yes	70/101 (69.3)*		35/48 (72.9)	47/60 (78.3)*
≥2 historical fractures				
No (reference category)	–	180/236 (76.3)	–	–
Yes		46/50 (92.0)		
History of hip fracture				
No (reference category)	–	–	233/282 (82.6)	–
Yes			12/13 (92.3)	
≥1 occurrence of immobility in the 12 months prior to enrollment				
No (reference category)	–	213/264 (80.7)	–	–
Yes		13/22 (59.1)*		
Previous PMO therapy in the 12 months before enrollment				
No (reference category)	–	–	61/81 (75.3)	–
Yes			184/214 (86.0)	
History of discontinuation of osteoporosis therapy				
No (reference category)	–	–	184/213 (86.4)	–
Yes			38/47 (80.9)	
N/A			23/35 (65.7)	
Reason for prescribing: failed other available osteoporosis therapy				
No (reference category)	269/375 (71.7)	–	128/162 (79.0)	–
Yes	142/178 (79.8)		117/133 (88.0)*	
Reason for prescribing: multiple risk factors for fracture				
No (reference category)	220/291 (75.6)	151/187 (80.7)	–	–
Yes	191/262 (72.9)	75/99 (75.8)		–
Reason for prescribing: intolerant to other osteoporosis therapy				
No (reference category)	–	–	198/234 (84.6)	–
Yes			47/61 (77.0)	

Where data are missing, the covariate did not enter the model for the specific country. *N1* is the number of patients in the 24-month FAS (*n* = 1479) with baseline covariate data for all selected covariates, and *n* is the number of persistent patients within the covariate group. Percentages are based on *N1*

*FAS* full analysis set, *N/A* not applicable, *PMO* postmenopausal osteoporosis

*p ≤ 0.05; **p ≤ 0.01; ***p ≤ 0.001

No covariate achieved the significance level required for entry into the model for all four countries. The only covariate to enter the model for three countries (Germany, Greece, and Belgium) was the occurrence of one or more falls in the 12 months before enrollment. Across these three countries, persistence rates were lower among patients who had had at least one fall in the 12 months prior to enrollment compared with those who had experienced no such falls. This association was statistically significant in Germany and Belgium (*p* values of 0.038 and 0.027, respectively) but not in Greece (*p* = 0.213). Further analysis of this covariate across all four countries revealed that patients with a fall in the 12 months before enrollment were generally older than those without a fall (49.8 vs 33.5%, respectively, were ≥75 years old, while 19.6 vs 29.7% were <65 years old). In addition, a greater proportion of those with a fall than without a fall had at least two chronic medical conditions (44.3 vs 33.1%. respectively). No other covariates entered the model for Belgium.

Modified Wolfe comorbidity index (MWCI) entered the model for Germany and Austria. In both countries, persistence rates were lower in patients whose MWCI was above the country-specific median (median = 1.0 for both countries) than in those whose MWCI was equal to or below the country-specific median. This association was statistically significant in Germany, but not in Austria (*p* = 0.048 and *p* = 0.054, respectively). The covariate “Reason for prescribing: multiple risk factors for fracture” also entered the model for Germany and Austria. In both countries, persistence rates were lower in patients for whom this reason was given than in those for whom this reason was not given; however, the association did not reach statistical significance for either country (*p* = 0.100 and *p* = 0.163, respectively).

The covariate “Reason for prescribing: failed other available osteoporosis therapy” entered the model for Germany and Greece. In Greece, persistence was higher in patients for whom this reason was given than in those for whom it was not given; this association was statistically significant (*p* = 0.033). A similar (non-significant) trend was seen in Germany (*p* = 0.126).

A number of covariates entered the model only for Greece. Trends towards lower persistence were seen for patients aged 75 years or older (vs those <65 years old), patients with a history of discontinuation of osteoporosis therapy (vs no history of discontinuation), patients with the prescribing reason of “intolerant to other osteoporosis therapy” (vs this reason not given), and for current and former smokers (vs not current or former smoker). The association was only significant for individuals aged 75 years or older and those with a current smoking status (*p* = 0.001 and *p* = 0.025, respectively). In contrast, history of hip fracture (vs no history of hip fracture), age 65–74 years (vs <65 years old), the number of concomitant medications being higher than the country-specific median (vs the same as the median or lower), and receipt of previous PMO therapy in the 12 months before enrollment (vs no previous PMO therapy in the 12 months before enrollment) were associated with higher persistence but did not reach statistical significance.

Having at least one occurrence of immobility in the 12 months before enrollment entered the model for Austria and was significantly associated with lower persistence with treatment compared with patients who had not experienced immobility (*p* = 0.019). Further analysis of this covariate across all four countries showed that individuals with an episode of immobility in the 12 months before enrollment were generally older than those without an episode of immobility (54.4 vs 34.8% were ≥75 years old, respectively, and 20.0 vs 28.8% were <65 years old). Furthermore, 84.8% of patients with an episode of immobility had at least one chronic medical condition and 55.2% were taking more than two concomitant medications. Having at least two historical fractures also entered the model for Austria and was associated with a trend towards higher persistence compared with patients who had not had two or more historical fractures (*p* = 0.053). Having any chronic medical condition entered the model for Germany and was significantly associated with higher persistence compared with not having a chronic condition (*p* = 0.007).

### Safety

Safety was assessed in the 1480 patients included in the SAS. Across the four countries, 2.3–6.9% of patients reported ADRs (Table 3). The most frequently reported group of ADRs were those affecting the musculoskeletal and connective tissue systems (0.3–2.1% of patients; Table 3); the most common of these was arthralgia (in 0–0.7% of patients; Table 3). Across countries, 0.3–1.7% of patients reported ADRs relating to skin and subcutaneous tissue disorders, including pruritus, erythema, and rash (Table 3). Thirty-two patients (2.2%) discontinued denosumab therapy because of ADRs, the most frequent of which were skin and subcutaneous disorders (*n* = 8), musculoskeletal and connective tissue disorders (*n* = 7), general disorders and administration site conditions (*n* = 6), and infections and infestations (*n* = 6). Three patients (0.2%) reported serious ADRs, with no fatal events. The serious ADRs were two cases of osteonecrosis of the jaw (ONJ) and one case of musculoskeletal pain (discussed below). No cases of hypocalcemia or anaphylaxis were reported in any country.

**Table 3:** Safety data at 24 months in the SAS

Outcome	Germany (*N* = 579)	Austria (*N* = 300)	Greece (*N* = 300)	Belgium (*N* = 301)
All adverse drug reactions	40 (6.9)	7 (2.3)	14 (4.7)	9 (3.0)
Leading to discontinuation of denosumab	21 (3.6)	5 (1.7)	3 (1.0)	3 (1.0)
Serious adverse drug reaction	2 (0.3)	0 (0.0)	0 (0.0)	1 (0.3)
Leading to death	0 (0.0)	0 (0.0)	0 (0.0)	0 (0.0)
Adjudicated positive ONJ	2 (0.3)	0 (0.0)	0 (0.0)	0 (0.0)
Osteoporotic fracture^a^	30 (5.2)	4 (1.3)	9 (3.0)	21 (7.0)
Adjudicated positive atypical femoral fracture	0 (0.0)	0 (0.0)	0 (0.0)	1 (0.3)
Most frequently reported adverse drug reactions				
Musculoskeletal and connective tissue disorders	12 (2.1)	1 (0.3)	2 (0.7)	4 (1.3)
Arthralgia	4 (0.7)	1 (0.3)	0 (0.0)	2 (0.7)
Osteonecrosis of the jaw	2 (0.3)	0 (0.0)	0 (0.0)	0 (0.0)
Pain in extremity	1 (0.2)	0 (0.0)	1 (0.3)	0 (0.0)
Arthropathy	0 (0.0)	0 (0.0)	0 (0.0)	1 (0.3)
Back pain	0 (0.0)	0 (0.0)	1 (0.3)	0 (0.0)
Growing pains	1 (0.2)	0 (0.0)	0 (0.0)	0 (0.0)
Muscle spasms	0 (0.0)	1 (0.3)	0 (0.0)	0 (0.0)
Musculoskeletal pain	0 (0.0)	0 (0.0)	0 (0.0)	1 (0.3)
Myalgia	1 (0.2)	0 (0.0)	0 (0.0)	0 (0.0)
Osteitis	1 (0.2)	0 (0.0)	0 (0.0)	0 (0.0)
Pain in jaw	1 (0.2)	0 (0.0)	0 (0.0)	0 (0.0)
Trismus	1 (0.2)	0 (0.0)	0 (0.0)	0 (0.0)
Skin and subcutaneous tissue disorders	10 (1.7)	1 (0.3)	4 (1.3)	1 (0.3)
Pruritus	5 (0.9)	0 (0.0)	0 (0.0)	1 (0.3)
Erythema	3 (0.5)	0 (0.0)	0 (0.0)	0 (0.0)
Pruritus generalized	1 (0.2)	0 (0.0)	2 (0.7)	0 (0.0)
Rash	1 (0.2)	1 (0.3)	1 (0.3)	0 (0.0)
Dry skin	0 (0.0)	0 (0.0)	1 (0.3)	0 (0.0)
Skin mass	0 (0.0)	0 (0.0)	1 (0.3)	0 (0.0)

Data are shown as *n* (%). Safety data were analyzed in the SAS (*N* = 1480)

*ONJ* osteonecrosis of the jaw, *SAS* safety analysis set

^a^Osteoporotic fractures were defined as all fractures excluding those of the skull, facial bones, mandible, metacarpus, finger phalanges, toe phalanges, and cervical vertebrae and those not associated with known high trauma severity (fall from higher than the height of a stool, chair, first rung on a ladder, or equivalent (>20 in. [51 cm]), or severe trauma other than a fall) and pathological fractures

ADRs of osteoporotic fracture (fragility fracture) were reported in 64 women (4.3%), including fractures of the lumbar spine, humerus, wrist, hip, ribs, and pelvis. One patient (0.1%) had an atypical femoral fracture. Two independently adjudicated cases of ONJ were reported, both in Germany (Table 3). Both individuals had risk factors for ONJ (previous bisphosphonate use, old age, or invasive dental procedures). One case resolved after approximately 1 year. The outcome of the other patient is unknown. A medical history was not available for the patient with serious musculoskeletal pain. This individual was hospitalized for bone pain and had concurrent non-serious pollakiuria.

## Discussion

A number of retrospective database studies have reported 2-year persistence data for denosumab (SC Q6M), with values ranging from 38 to 62% [13–15]. In German and Swedish studies, persistence with denosumab was substantially higher than that seen with intravenous or oral bisphosphonates [13, 14]; in a Hungarian study, persistence was considerably lower with daily, weekly, and monthly treatments (10–18%) than with 6-monthly denosumab [15].

Our prospective study reports real-world persistence data with denosumab over the same period, ranging from 75.1 to 86.0% across the countries studied. This represents only a small change from the 12-month interim analysis reported previously, in which persistence with denosumab at 12 months was 87.0─95.3% [31]. The high level of persistence observed over 24 months was supported by a high MCR (87.4–92.4%) and high rates of adherence (62.9–70.1%; lower than persistence owing to the more stringent definition). Of note, persistence levels were similar across all four countries studied, regardless of different cultures, healthcare regulations, and geographic distribution. In addition, the safety profile of denosumab was consistent with that in previously published reports [23–27, 36, 37].

Differences between our data and those of others may be, in part, due to the prospective design of our study. The other studies discussed above were retrospective; hence, the recording of persistence may have been more rigorous in our study. Furthermore, owing to the prospective design of this study, patients were aware that their medication-taking behavior was being observed and may have modified their behavior towards being more persistent with treatment [38]. Indeed, another prospective study of patients in Austria found similarly high levels of persistence with denosumab at 24 months (83.0%) [34]. This suggests that interventions monitoring patients’ medication-taking behavior may have a positive impact on persistence. Strategies to improve persistence proposed on the basis of our data could be complemented by combining each dose/injection with simple biochemical tests to measure response (e.g., by monitoring bone turnover markers), which may emphasize the importance of treatment to patients. Indeed, this intervention has been shown to improve persistence in individuals with good responses to treatment [39].

When reporting the multivariate analysis of 24-month persistence, we selected covariates which we deemed clinically relevant for discussion in this article. A number of covariates which we did not consider clinically relevant demonstrated statistically significant associations with 24-month persistence (e.g., reminder service available, academic center); these are detailed in Online Resource 1. Of note, other studies have reported that calcium and vitamin D treatment influences persistence [14, 40]. However, owing to the over-the-counter accessibility of these medications and the reliance on patient recall, these data may not be adequately robust and we did not deem them to be clinically relevant covariates. Given the small number of patients who were non-persistent in our study, a high proportion of them would need to have the same characteristic for statistical significance to be reached; hence, data from the multivariable analysis should be interpreted with caution. Despite this limitation, several covariates were significantly associated with persistence in at least one country.

Contrary to expectations, patients with falls in the 12 months before enrollment were less likely to be persistent with treatment than those without falls; this association was seen in three of the four countries studied (Germany, Greece, and Belgium). These data contrast with findings from a UK study of persistence, in which a fall before starting treatment was associated with improved compliance [16]. It is worth noting that the latter study classified patients as being persistent if they had received at least one bisphosphonate prescription in each 12-month period during follow-up. These data suggest that physicians in the UK may be more likely than those in other countries to prioritize osteoporosis treatment following a fall. In order to try to explain our finding, we examined the characteristics of patients with and without falls in the 12 months before enrollment. It was noted that compared with patients who had not had a fall, those with a history of falls tended to be older, with a greater likelihood of having at least two chronic medical conditions. Although age and the presence of a chronic medical condition were not individually associated with persistence across the same three countries, we might speculate that the various characteristics of the patients with a fall history interacted to generate a patient profile with a tendency for low persistence.

In common with other studies showing that increased comorbidity is associated with lower persistence [16, 18, 20], patients with a high MWCI were less likely to be persistent with therapy than those with a lower MWCI. Individuals with multiple comorbidities are likely to have a high medication burden, which may be confusing and could result in osteoporosis treatment being considered a low priority. In addition, when attempting to reduce medication burden, physicians and patients may deprioritize osteoporosis therapy. Patients who were prescribed denosumab because their previous osteoporosis therapy failed were more likely to be persistent than those who were not prescribed denosumab for this reason. Such patients may be better educated regarding their disease and the need for persistence than patients who have not previously failed treatment, perhaps as a result of discussions with physicians at the time of previous treatment failure. In addition, patients for whom bisphosphonate treatment was unsuccessful may see denosumab as a last chance. This association was also seen in the Swedish database study of persistence discussed previously [14].

A history of the presence or absence of prior fractures is known to be associated with higher persistence [17]; however, this variable only entered the model for Austria. Because the risk of future fracture increases substantially with the number of previous fractures, we used a cut-off level of at least two previous fractures to increase the discrimination between groups for this covariate. Furthermore, previous fractures could include fragility fractures; hence, the higher threshold increases the probability that patients in the fracture group had experienced fragility fractures. In contrast, also in Austria, having an episode of immobility in the 12 months before enrollment was associated with lower persistence than not having immobility in the same period. This could be because patients experiencing immobility are likely to be older and frailer than those who do not have periods of immobility.

The age group covariate was only eligible for entry into the multivariable model in Greece. Patients aged 75 years or older had significantly lower persistence with treatment than those aged less than 65 years, whereas there was a trend towards higher persistence in individuals aged 65–74 years. Greek treatment guidelines recommend that, in the absence of other risk factors, BMD measurements should be carried out when patients reach 65 years of age; at this point, disease awareness (and therefore persistence) is likely to be higher than in younger individuals [41]. Patients older than 75 years may have more chronic conditions and more concomitant medications and may therefore deprioritize osteoporosis treatment. In addition, individuals older than 75 years may be more likely to forget to book an appointment. Reports in the literature also vary; while the results of many studies suggest that young age is associated with lower persistence [16, 18, 19], an investigation by Lekkerkerker et al. suggested that persistence declines with age [17]. Therefore, there may be a window of age during which persistence is optimal.

Our finding that current smokers have lower persistence than non-smokers is in line with previously published data [21], suggesting that such individuals may be less likely to heed health advice than non-smokers.

The facts that no clinically relevant covariates entered the model for all four countries, that only one entered the model for three countries, and that the majority of covariates were not significantly associated with persistence suggest that persistence with denosumab therapy is consistently high across patient populations. This could partly be responsible for the higher BMD increases achieved with denosumab than with other antiresorptives. Furthermore, in most countries, factors known to be associated with increased fracture risk, such as age, smoking status, and previous fracture, were not associated with persistence. This suggests that persistence with denosumab is generally high, even in patients at high risk of fracture. The exception was the covariate “≥1 fall in the 12 months prior to enrollment,” which is associated with an increased risk of fracture [42] but, in our study, was associated with low persistence. Improving persistence in this patient group therefore has the potential to have a considerable impact on fracture rates.

The lower impact of patient characteristics on persistence with denosumab treatment observed in the current study compared with that in persistence studies of bisphosphonates may have resulted, in part, from the availability of the long-acting injectable formulation. Long-acting injectables are generally administered by healthcare providers rather than being self-administered; hence, patients are less likely to be able to deviate from the prescribing instructions. Furthermore, longer dosing intervals between treatments may be preferred by patients over more frequent dosing regimens [15, 29, 30, 43].

As reported previously, this study had some limitations [31]. Physicians actively agreed to participate in the study and came from centers that could support their participation; therefore, they may not be representative of all physicians and sites treating women with PMO in Germany, Austria, Greece, and Belgium or other countries. The study enrolled a high proportion of patients who had previously received another osteoporosis medication. This is likely to reflect the higher proportion of patients in real-world practice who have previously been treated for osteoporosis compared with those who are newly diagnosed. It could also be a reflection of country-specific requirements in which patients are eligible for denosumab treatment.

MCR as a measure of medication-taking behavior has not been widely used; however, MCR is similar to the more commonly used medication possession ratio (MPR), in that both describe the proportion of time during which patients have access to a medication. MPR is well suited to assessment of oral medications, as it indicates the proportion of days on which the patient had an adequate supply of medication. It is less appropriate, however, for long-acting injectable medications, with which a patient is covered therapeutically for a certain amount of time [44]. MCR reflects the proportion of days that a patient is covered with respect to the medication over a given interval after receiving the injection and is the more appropriate tool for evaluating injectable medications [31, 45].

Differences in reimbursement criteria across countries may also have influenced the data; for example, in Austria, denosumab was only used at second line. However, comparison of patient baseline characteristics showed that the proportion of patients with previous osteoporosis treatment was not considerably higher in Austria than in the other countries studied, probably because denosumab had recently been approved in Europe. The high proportion of patients who had previously received another osteoporosis medication also suggests that the women enrolled in this study may have had more severe disease than the overall osteoporosis population. Furthermore, although enrollment did not start until approximately 1 year following the availability of denosumab in Europe, participating physicians may have been early users of the therapy and could have therefore selected denosumab as a treatment for patients with chronic or severe disease. Additionally, patients may have switched to denosumab treatment following intolerance or poor adherence to bisphosphonates. Overall, patient characteristics were similar across countries. It would therefore be interesting to extend this comparison to countries in which these variables are likely to be more diverse. Another consideration is that the study was observational and we were unable to assess the impact of persistence on fracture risk. With regard to the multivariable analysis of persistence, and as noted previously, it was difficult to identify any covariates that could be used in clinical practice to improve persistence further. Finally, this study did not assess the number of patients who refused osteoporosis medication at first prescription (primary non-adherence). This was outside the scope of our study but is nonetheless an important aspect of medication-taking behavior.

## Conclusions

The results of this study show that persistence with denosumab therapy remains high at 24 months, regardless of baseline covariates, in patients at high risk of fracture, across all four European countries studied. The consistently high level of persistence across the countries indicated that it was relatively insensitive to differences in culture, healthcare regulations, and geographic distribution. While acknowledging the limitations of comparing data from studies of different designs, persistence in this study appeared higher than that previously observed with bisphosphonates, with treatments requiring daily, weekly, or monthly dosing, and, indeed, higher than in that found in other studies of denosumab. Furthermore, baseline patient characteristics did not appear to have a considerable effect on persistence, suggesting that medication-taking behavior with denosumab is good in a broad range of patients. Further studies are needed to enable better characterization of the predictors of low persistence.

## Electronic supplementary material

ESM 1 (PDF 408 kb)
