# Supplementary material for: Factors associated with high 24-month persistence with denosumab: results of a real-world, non-interventional study of women with postmenopausal osteoporosis in Germany, Austria, Greece, and Belgium
Source: Arch Osteoporos. 2017 Jun 22;12(1):58. doi: 10.1007/s11657-017-0351-2 (PMC5486684; doi:10.1007/s11657-017-0351-2)
Supplement: Supplementary file 1 — (PDF 408 kb) [file 11657_2017_351_MOESM1_ESM.pdf]

Factors associated with high 24-month persistence with denosumab: results of a real-world, non-interventional study of women with postmenopausal osteoporosis in Germany, Austria, Greece, and Belgium

A. Fahrleitner-Pammer[1] · N. Papaioannou[2] · E. Gielen[3] · M. Feudjo Tepie[4] · C. Toffis[5] · I. Frieling[6] · P. Geusens[7] · P. Makras[8] · E. Boschitsch[9] · J. Callens[10] · A. D. Anastasilakis[11] · C. Niedhart[12] · H. Resch[13] · L. Kalouche-Khalil[14] · P. Hadji[15]

[1]Department of Endocrinology and Metabolism, Medical University Graz, Graz, Austria

[2]University of Athens, Medical School, Laboratory for the Research of Musculoskeletal System, KAT Hospital, Athens, Greece

[3]Department of Geriatrics and Center for Metabolic Bone Diseases, UZ Leuven, Leuven, Belgium

[4]Amgen Ltd, Uxbridge, UK

[5]Amgen Ltd, Cambridge, UK

[6]Osteoporosis Center, Hamburg, Germany

[7]Department of Internal Medicine, Maastricht University Medical Centre, Maastricht, Netherlands and University Hasselt, Diepenbeek, Belgium

[8]Department of Endocrinology and Diabetes, 251 Hellenic Airforce and VA General Hospital, Athens, Greece

[9]Ambulatorium KLIMAX, Menopause and Osteoporosis Clinic, Vienna, Austria

[10]Department of Orthopedics, Rheumatology and Physical Medicine and Rehabilitation, AZ Zeno Knokke, Belgium

[11]Department of Endocrinology, 424 General Military Hospital, Thessaloniki, Greece

[12]Osteoporosis Center, Heinsberg, Germany

[13]Medical Department II, St Vincent Hospital, Academic Teaching Hospital of the Medical University of Vienna, Vienna, Austria and Medical Faculty, Sigmund Freud University, Vienna, Austria

[14]Amgen (Europe) GmbH, Zug, Switzerland

[15]Department of Obstetrics and Gynecology, Krankenhaus Nordwest, Frankfurt, Germany

Corresponding author:

P. Hadji

Department of Obstetrics and Gynecology, Krankenhaus Nordwest, Frankfurt, Germany

Journal: *Osteoporosis International*

Electronic Supplementary Material

Online Resource 1 Covariates entered into the multivariable analysis model and statistically significant associations with 24-month persistence

**Covariate**	**Country**			
	**Germany**	**Austria**	**Greece**	**Belgium**
**Age**			$p < 0.0001$	
**Currently smoking**			$p = 0.0246$	
**Formerly smoking**			$p = 0.1776$	
**Modified Wolfe comorbidity index**	$p = 0.0481$	$p = 0.0541$		
**Any chronic medical condition**	$p = 0.0065$			
**Number of concomitant medications taken at baseline**			$p = 0.1596$	
**≥1 fall in the 12 months prior to enrollment**	$p = 0.0381$		$P = 0.2133$	$p = 0.0269$
**≥2 historical fractures**		$p = 0.0525$		
**History of hip fracture**			$p = 0.0838$	
**≥1 occurrence of immobility in the 12 months prior to enrollment**		$p = 0.0193$		
**Previous PMO therapy in the 12 months before enrollment**			$p = 0.0751$	
**History of discontinuation of osteoporosis therapy (not calcium or vitamin D)**			$p = 0.1940$	
**Reason for prescribing: failed other available osteoporosis therapy**	$p = 0.1259$		$p = 0.0330$	
**Reason for prescribing: intolerant to other osteoporosis therapy**			$p = 0.1063$	
**Reason for prescribing: multiple risk factors for fracture**	$p = 0.0999$	$p = 0.1633$		
Reminder service available		$p = 0.0179$	$p = 0.0058$	
Academic center	$p = 0.0105$	$p = 0.0038$		$p = 0.0015$
Employment status			$p = 0.1495$	
Center type	$p = 0.0681$			
Proximity to clinic (minutes)				$p = 0.1266$
Region	$p = 0.1721$			
Cause of menopause		$p = 0.1119$		
Physician sex		$p = 0.1320$	$p = 0.0491$	
Physician specialty	$p = 0.0991$	$p < 0.0001$		$p = 0.0492$
Physician years of practice		$p = 0.0161$	$p = 0.0580$	
Prior calcium and/or vitamin D supplements		$p = 0.1047$	$p = 0.0610$	

P values show the statistical significance of the covariate in the country-specific model, with *p* values <0.05 considered to be significant (shown underlined). Covariates in bold text were considered to be clinically relevant

PMO postmenopausal osteoporosis

Key shown below

(black)	Covariate not eligible for inclusion in the country-specific model	(green)	Covariate significantly associated with 24-month persistence
(grey)	Covariate not significantly associated with 24-month persistence		

Online Resource 2 MCR of denosumab at 24 months

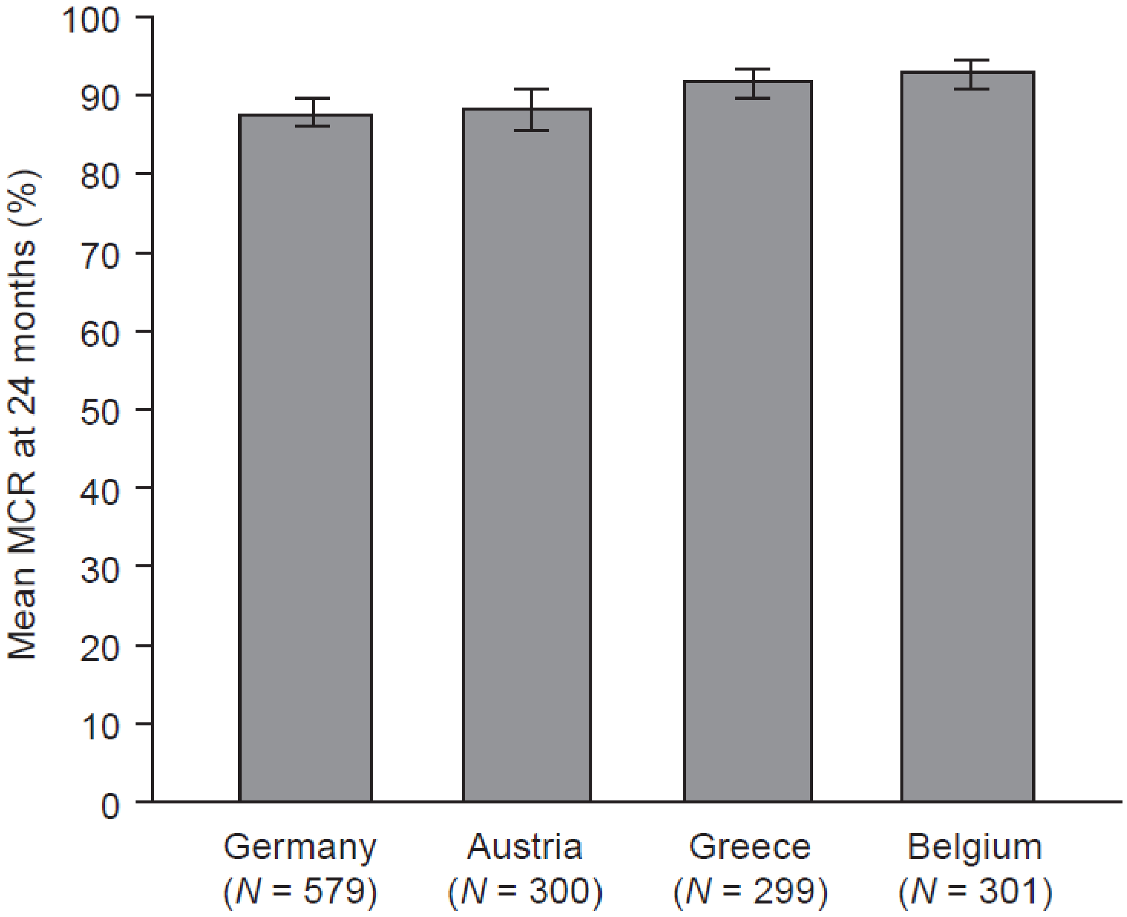

Data are shown as mean percentages ± 95 % CIs

The MCR was calculated as the percentage of time that a patient was covered by denosumab, as assessed from prescription records, and was based on the assumption that each injection of denosumab provides 6 months of medication coverage

CI confidence interval, *MCR* medication coverage ratio

Online Resource 3 Sensitivity analysis of persistence and adherence with denosumab at 24 months

	Germany<br>(*N* = 579)	Austria<br>(*N* = 300)	Greece<br>(*N* = 299)	Belgium<br>(*N* = 301)
Persistence, %				
6 months + 4 weeks	65.3 (61.3–69.2)	69.3 (63.8–74.5)	72.6 (67.1–77.6)	76.4 (71.2–81.1)
6 months + 6 weeks	72.2 (68.4–75.8)	78.0 (72.9–82.6)	79.3 (74.2–83.7)	81.7 (76.9–85.9)
6 months + 12 weeks	77.4 (73.7–80.7)	82.0 (77.2–86.2)	86.6 (82.2–90.3)	87.7 (83.5–91.2)
Adherence, %				
6 months ± 6 weeks	71.2 (67.3–74.8)	77.0 (71.8–81.6)	78.6 (73.5–83.1)	79.7 (74.7–84.1)
6 months ± 8 weeks	74.8 (71.0–78.3)	79.7 (74.7–84.1)	81.9 (77.1–86.1)	85.0 (80.5–88.9)
6 months ± 12 weeks	77.2 (73.6–80.6)	82.0 (77.2–86.2)	86.6 (82.2–90.3)	87.0 (82.7–90.6)

Data are shown as percentages (95 % confidence intervals)
